# The history of surgery and surgical training in the UK

**DOI:** 10.12669/pjms.37.5.4628

**Published:** 2021

**Authors:** Thomas Payne, Joseph D. Toms, Ahsan Zaidi, Sri G. Thrumurthy

**Affiliations:** 1 Thomas Payne MBChB, MSc, Clinical Teaching Fellow, St George’s Hospital NHS Foundation Trust, London, UK; 2 Joseph D. Toms MBBS, BSc, Trust Doctor in General Surgery, Epsom and St Helier Hospitals NHS Trust, London, UK; 3 Ahsan Zaidi MBBS MS LRCP FRCS, Consultant General and Upper GI Surgeon, Epsom and St Helier Hospitals NHS Trust, London, UK; 4 Sri G. Thrumurthy MBChB (Hons) MRCP (UK) FRCS (Gen Surg) FHEA FAcadMEd Advanced Endosurgery Fellow, King’s College Hospital NHS Foundation Trust, London, UK

**Keywords:** Surgery, Training, UK, History

## Abstract

Surgery has a rich history, and in order to understand the various training pathways for aspiring surgeons one must have an appreciation of the evolution of surgery. This manuscript aims to deliver a brief review of the history of surgery, and explore the historical moments that have shaped the training pathway of surgeons in the United Kingdom (UK), and in doing so disseminate the latest information about surgical training in the UK.

## A brief history of surgical practice

The word surgery originates from the Greek translation of *kheirourgía*, meaning “hand work”, referring to the branch of medicine responsible for the physical manipulation of bodily structures.

Some of the earliest evidence of dedicated surgical procedures were discovered by Paul Broca, where during his anthropological work he concluded that trepanation of the skull had been performed as far back as 7000 years ago.^1^ It is possible of course that rudimentary surgery was being performed before this time on battlefields across the world, since war is as old as human history, and where there are injured soldiers, there are caregivers.^2^ This practice was initially driven by desperation rather than by any knowledge or experience of outcomes or techniques, and often performed by the patient themselves. The Romans practiced relatively modern military medicine with dedicated field hospitals, set up in such a way as to protect their water supply, with latrines located downstream, suggesting a knowledge of sanitation and its impact on healthcare.

However, until the Industrial revolution began in 1760 the evolution of surgery was limited by bleeding, infection and pain. John Hunter was a renowned surgeon of the 18^th^ century who never completed a recognised course of study, or even a degree in medicine, yet is acknowledged as the founder of pathological anatomy and by many as the father of modern scientific surgery.^3^ He served during the seven years’ war (1756 - 1763) where his experiences lead to a publication of a treatise on gunshot wounds.^4^

The creation of the Company of Barber-Surgeons of London in 1540 marks the first official record of an organized surgical profession and was a milestone in the history of English surgery. Services provided included bloodletting (symbolised by the white background and red stripe of the traditional barber’s pole), dental extractions and lancing abscesses. This company was responsible for teaching and training apprentices, providing licenses for practice and appointing surgeons to the army. The institution came under pressure from the medical profession, and eventually resulted in the formation of a Company of Surgeons in 1745, which gained a royal charter in 1800 to become the Royal College of Surgeons.^5^ Some remnants of this history remain within the surgical profession today, where UK surgeons adopt the title of ‘Mr’, shedding their former ‘Dr’ once they become members of the royal college.

The first transfusion of blood was recorded in England in 1818 by James Blundell, who also probably performed the first hysterectomy for cancer later in 1828,^6^ although many sources suggest the first hysterectomy was performed by Charles Clay in Manchester, England, in 1843,^7^ the same year that ether was first used as an anaesthetic agent.^8^ Following the introduction of anaesthesia in the mid-19th century, surgery was no longer required to be accelerated to the same extent in order to limit duration and associated pain. It also enabled surgery to progress from amputation and external excisions to internal regions of the body. Joseph Lister’s work^9^ built upon earlier observations by Semmelweis,^10^ and in the 1860s light was being shed on the causes of post-operative infection. The use of antiseptic and subsequently aseptic techniques was introduced. For his work in this area and the advances made possible by it, Lister is often referred to as the father of modern surgery.

The next advance in the surgical field occurred at the end of the 19th century with the discovery of X-ray imaging by Wilhelm Roentgen. The use of X-rays was accepted almost globally as useful in medical diagnosis, but it was not for at least a decade until its use was widespread in patient care.^11^ In the 1970s advances in computerised technology led to the discovery of cross-sectional X-ray imaging. The use of computerised tomography enabled much more accurate surgical diagnosis and planning. Availability and techniques have improved more recently (with less radiation, for example) allowing for a wider practice of pre-operative being imaging adopted by many surgical specialties. The option of cross-sectional imaging is reshaping surgical management, even today. One such example includes the ongoing controversary over CT scanning before diagnostic laparoscopy in cases of suspected appendicitis.^12^

The 20^th^ century saw additional advances including electrosurgery, endoscopy, computer assisted surgery, laser surgery and robotic surgery. Electrosurgery is now a hallmark of modern operating and incorporated into the curriculum of most surgical trainees, whereas robotic surgery remains a specialist area that is only recently becoming a new standard of care.^13^ Laser surgery began in the 1980s and was rapidly adopted as a new technique for ablation, particularly in cancers of the upper aerodigestive tract.^14^ 1990 saw the laparoscopic revolution and an end to the surgical dogma that ‘large problems require large incisions’.^15^

The 21^st^ century has much to bring to the field of surgery. The UK Royal College of Surgeons (RCS) has commissioned a report into the future of surgery which has suggested some of the technological advancements that will revolutionise surgery in the coming decades. These include the widespread use of robot-assisted surgery, artificial intelligence (AI) software aiding in diagnosis and intervention, and the implementation of 3D bio-printing of tissues and organs, perhaps eliminating the risks of transplant rejection.

## Surgical training in the UK

Surgical training has mirrored the evolution of surgery in many respects. As technological advancements are made, training must adapt to reflect those changes in practice, ensuring the next generation of surgeons are up to date with modern techniques.

Apprentice style surgical training began as far back as the 16^th^ century, and has continuously evolved and grown, particularly rapidly in the last 30 years. From an informal on-the-job training pathway, where self-evaluation, mentor approval and surgical demand promoted progress (in what was at the time more of a trade or craft) to the current domain-assessed, streamlined process.

The apprenticeship-based structure was updated, following the formation of the royal college, to require fellowship of the royal college of surgeons (FRCS), attained by passing structured exams. Technical skills continued to be evaluated by senior supervising surgeons but training occurred in departments with recognised mentors and senior specialists.

In 1993 the system for surgical training in the UK underwent significant alterations under the supervision of Sir Kenneth Calman, chief medical officer for England. This resulted in an annual review of competency progression (ARCP) and inclusion of a logbook to enable review of surgical experience. The initial aim of these ‘Calman reforms’ was to streamline the progression of middle grades in order to produce clinically competent consultants much earlier than in previous years.^16^ Satisfactory completion of these elements enabled progression to the next year of training. It is worth noting that these reforms applied to every medical specialty, and were not unique to surgical training. The Calman formula was later complemented by the inclusion of an operative competency form to supplement the surgical logbook.

In 2005 the surgical training programme was transformed by further national reforms to junior doctor training. The system was termed Modernising Medical Careers (MMC) which constituted a 2-year foundation training programme followed by specialty training. Assessment was by means of a record of in training assessment (RITA). Following this update, the training programme was amended to comply with the European Working Time Directive (EWTD) which, in theory, reduced the number of surgical training hours from approximately 20,000 to 11,520 for each trainee.^17^ Again, the MMC system was applied nationally across all training pathways, and was not specific to surgery. This has resulted in a standardised and uniform progression from medical school to specialty training. All junior doctors receive a similar experience during their foundation years, and must achieve an ARCP at the end of each year for satisfactory completion.

In 2007 the Intercollegiate Surgical Curriculum Programme (ISCP) was introduced in an attempt to streamline the training process across sub specialties by including competence-based training along with regular, standardised assessment at Foundation level as well as at the beginning, middle and end of specialty training, across four domains. These domains are: knowledge, judgment, technique and professionalism.^18^ Trainee evaluation occurs at ARCP including a review of Work Place Based Assessments (WPBA), such as mini Clinical Evaluation Exercises (Mini-CEX), Case-Based Discussions (CBD) and Direct Observation of Procedural Skills (DOPS).

**Fig.1:**
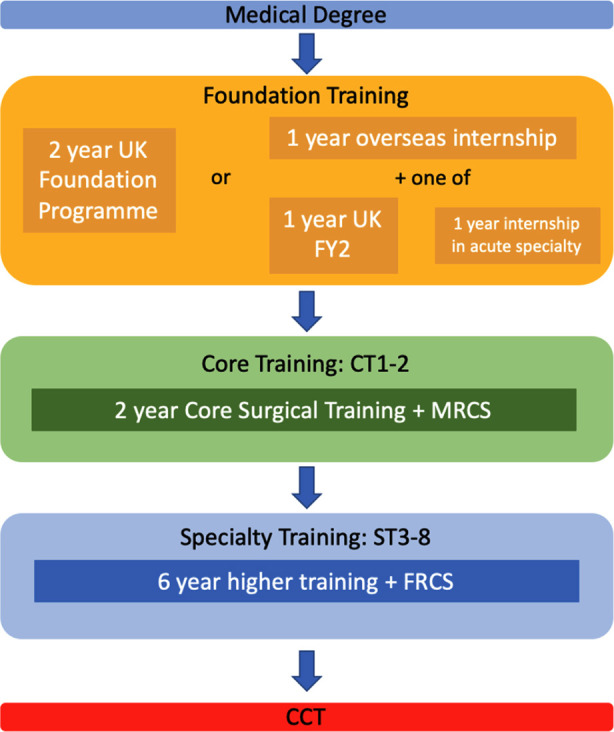
Current pathway for non-run-through surgical training

Entering a surgical career at present involves either appointments through fixed-term positions or ‘staff grade’ roles in surgical jobs, or via the training pathway, which is by far the more direct route. This begins after Foundation training (two years) by entering Core Surgical Training (two years), followed by Specialty Training (six years, beginning with the denomination ‘ST3’, after CT 1 and 2), before completion of training (by achieving the certificate of completion of training, CCT). After this, surgeons may apply for a consultant post. There are opportunities for juniors to enter specialty training directly after completion of foundation training at ST1 level, known as run-through training, but this is limited to certain specialties such as cardiothoracic surgery, neurosurgery and recently piloted in 2018 for otolaryngology (ENT).^19^ The run-through scheme is popular, and competitive, amongst applicants as it removes the need to apply for ST3 after core training, and suits those individuals who have a certain specialty in mind from an earlier point in their career, however it is considered by some to be an easier route into specialty training, resulting in a lower quality candidate at the ST3 level. Fig.1, 2 illustrate the differences between the traditional training route and run-through schemes.

**Fig.2:**
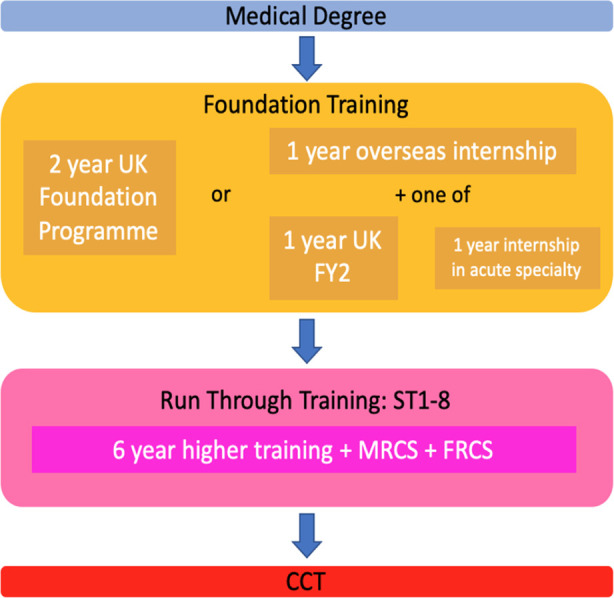
Current pathway for run-through surgical training.

Introduction of the Improving Surgical Training (IST) programme has been the latest change to UK surgical training at participating trusts.^20^ The pilot was commenced in 2018 and comprises 60% training time with protected feedback and reflection time, retaining 40% for service provision. It is a joint project between the Royal College of Surgeons of England (RCSEng) and Health Education England (HEE), created in response to the 2013 Shape of Training report by the General Medical Council (GMC).^21^ The project is an evidence-based scheme designed to improve job satisfaction amongst trainees by providing more support and protected training time, and inclusion of more technology-driven and simulation-based learning to enhance education. IST began solely for general surgery trainees, but has since expanded to include vascular surgery, urology and trauma and orthopaedics. The project is closely monitored by the GMC and an independent company in order to evaluate whether or not it does improve surgical training, although it currently represents an exciting opportunity for potential applicants.

## CONCLUSION

Surgical training varies greatly between countries and different health services. A universal feature is its competitiveness, although in the United States (US) for example matching in surgical residency is particularly challenging. This is in part due the number of applicants, resulting in higher competition ratios. The competition ratio to enter into Core Surgical Training in the UK is approximately 4:1, although this differs each year. The difference between these two nations is likely a consequence of the national shortage of UK doctors. It is possible, however, that this will change as we move into a post-COVID-19 expansion of health care.
